# Median raphe region stimulation alone generates remote, but not recent fear memory traces

**DOI:** 10.1371/journal.pone.0181264

**Published:** 2017-07-14

**Authors:** Diána G. Balázsfi, Dóra Zelena, Lívia Farkas, Kornél Demeter, István Barna, Csaba Cserép, Virág T. Takács, Gábor Nyíri, Flóra Gölöncsér, Beáta Sperlágh, Tamás F. Freund, József Haller

**Affiliations:** 1 Department of Behavioral Neurobiology, Institute of Experimental Medicine, Budapest, Hungary; 2 János Szentágothai School of Neurosciences, Semmelweis University, Budapest, Hungary; 3 Department of Cellular and Network Neurobiology, Institute of Experimental Medicine, Budapest, Hungary; 4 Department of Pharmacology, Institute of Experimental Medicine, Budapest, Hungary; Technion Israel Institute of Technology, ISRAEL

## Abstract

The median raphe region (MRR) is believed to control the fear circuitry indirectly, by influencing the encoding and retrieval of fear memories by amygdala, hippocampus and prefrontal cortex. Here we show that in addition to this established role, MRR stimulation may alone elicit the emergence of remote but not recent fear memories. We substituted electric shocks with optic stimulation of MRR in C57BL/6N male mice in an optogenetic conditioning paradigm and found that stimulations produced agitation, but not fear, during the conditioning trial. Contextual fear, reflected by freezing was not present the next day, but appeared after a 7 days incubation. The optogenetic silencing of MRR during electric shocks ameliorated conditioned fear also seven, but not one day after conditioning. The optogenetic stimulation patterns (50Hz theta burst and 20Hz) used in our tests elicited serotonin release *in vitro* and lead to activation primarily in the periaqueductal gray examined by c-Fos immunohistochemistry. Earlier studies demonstrated that fear can be induced acutely by stimulation of several subcortical centers, which, however, do not generate persistent fear memories. Here we show that the MRR also elicits fear, but this develops slowly over time, likely by plastic changes induced by the area and its connections. These findings assign a specific role to the MRR in fear learning. Particularly, we suggest that this area is responsible for the durable sensitization of fear circuits towards aversive contexts, and by this, it contributes to the persistence of fear memories. This suggests the existence a bottom-up control of fear circuits by the MRR, which complements the top-down control exerted by the medial prefrontal cortex.

## Introduction

Due to its links to anxiety and post-traumatic stress disorder, the way in which adverse experience is transformed into persistent fear memories is a fundamental question in neuroscience and psychiatry. Preclinical studies making use of the conditioned fear model implicate effector brain regions, such as the amygdala and periaqueductal gray in the etiology of dysfunctional fear states [1, 2]. The stimulation of these brain areas can elicit fear-like behavior (e.g. freezing) even in the absence of traumatic stress or fear learning, and the very same regions undergo plastic changes in response to traumatic stress, which appear causally linked to the development of learned fear. While such studies identify a basic mechanism of fear learning, the neural mechanisms that induce neuronal plasticity in these regions are poorly understood.

The serotonergic nuclei of the brainstem are good candidates for plasticity induction in response to stress, as they are activated by noxious stimuli, and induce plastic neural changes that may underlie fear expression [3–5]. Moreover, serotonergic nuclei also host glutamatergic mechanisms [6, 7], which are implicated in brain plasticity as well [8, 9]. In fact, earlier studies showed that the median raphe region (MRR) rather than the dorsal raphe (DRN) have a role in this process. Particularly, both the neurochemical inhibition, and the destruction of the MRR inhibited contextual conditioned fear elicited by electric shocks, whereas DRN mechanisms had disparate effects on the same response [10–13]. Recent studies provided further details regarding the impact of MRR on conditioned fear. For example it was recently demonstrated that the optogenetic stimulation of MRR regulates hippocampal ripple activity, which has a role in the consolidation of fear memory; particularly, photostimulation of the MRR immediately after shock-conditioning inhibited conditioned fear responses 24h later [14]. Studies performed in zebrafish showed that the role of the MRR in fear learning extended to active responses (e.g. avoidance learning) as opposed to passive responses such as conditioned freezing [15]. Albeit these and similar studies do show that MRR contributes to the encoding and expression of conditioned fear responses elicited by stressful events, usually electric shocks, no earlier study inquired into its direct role, which–as we hypothesize here–may go beyond the modulation of fear learning elicited by other brain areas.

The premises of our hypothesis are as follows: (i) noxious stimuli–including those that are used as unconditioned stimuli in fear learning paradigms–strongly activate the MRR, (ii) the electrical stimulation of MRR induces either behavioral freezing or an unnatural forced movement, suggesting that the stimulation is aversive, and (iii) MRR hosts mechanisms that contribute significantly to fear-induced neural plasticity [3, 4, 6–9, 16]. Based on these we hypothesized that MRR may *per se* elicit recent and/or remote fear responses.

To test this hypothesis, we substituted electric shocks with channelrhodopsin (ChR2)-mediated optic stimulation of MRR in a fear conditioning-like paradigm ("MRR-conditioning") and tested subsequent fear responses over a week. The optic stimulation activated upstream brain areas were evaluated by c-Fos immunohistochemistry. Halorhodopsin (NpHR)-mediated silencing of the MRR during electric shock-conditioning was also used to test whether the effects of MRR stimulation are congruent with the role of this structure in classical fear conditioning.

## Materials and methods

### Animals

We studied adult C57BL/6N male mice (Charles River, Budapest, Hungary) housed individually under a standard 12h light–dark cycle (lights on at 6 am), with food and water available *ad libitum*. Experiments were approved by the local committee for animal health and care (Animal Welfare Committee of the Institute of Experimental Medicine) and performed according to the European Communities Council Directive recommendations for the care and use of laboratory animals (2010/63/EU).

### Virus injection and optogenetics

For the optical control of the MRR, 40 nL adeno-associated virus vector (AAV; Penn Vector Core, PA, USA) encoding ChR2 (AAV2.5.hSyn.hChR2(H134R)eYFP.WPRE.hGH; 1.3e12 GC/ml; Addgene26973) or NpHR (AAV9.hSyn.eNpHR3.0-eYFP.WPRE.hGH; 2.04e12 GC/ml; Addgene26972) were injected into the MRR from glass pipettes (tip diameter 20–30 μm) connected to a MicroSyringe Pump Controller (World Precision Instruments, Sarasota, FL, USA) under deep anaesthesia (intraperitoneal injection of 25mg/kg xylazine and 125mg/kg ketamine in 0.9% NaCl). The coordinates were the followings: -4.10 mm from Bregma; 0.0 mm lateral to midline and -4.60 mm ventral to the skull. Two weeks after the injection mice were implanted with optic fibers (diameter: 250 μm; flat tip) 10° from dorsal at a coordinate -4.80 mm from Bregma; 0.0 mm lateral to midline and -4.1 mm ventral to the skull. Optic fibers for implantation and light stimulation were custom made from multimode optical fiber (AFS 105/125Y, NA: 0,22, low-OH, Thorlabs Corp., Munich, Germany) and flanged zirconia ferrule (LMFL-172-FL-C35-OSK, Senko, Hampsire, UK). Implants were secured by screws and acrylic resin (Duracryl Plus; SpofaDental, Czech Republic). Behavioral experiments started after 4–7 days recovery. Laser beams (473-nm for ChR2, and 561-nm for NpHR activation) were generated by low noise diode-pumped solid-state lasers (Ikecool Corp., Anaheim, CA, USA), then collimated and guided to the implanted optic fiber by fiber-optic patch cords (FT900SM and FT030-BLUE, Thorlabs Corp.). Net energy output was measured by laser power meter (Coherent, LaserCheck, Santa Clara, CA, USA) before and after the experiments. Data were used only when optic fibers transferred 10–20 mW net energy at continuous light emission. After behavioral studies, mice were perfused and histological analysis was performed.

### Anatomical analysis

After the experiments, mice were deeply anaesthetized (see above) and transcardially perfused with 0.1M phosphate buffered saline (PBS) for 1 minute, then with 4% (w/v) paraformaldehyde in PBS for 20 minutes. Optic fibers were carefully removed, brains were taken out, and post-fixed for 24 hours in fixative at +4°C. Brains were cryo-protected by 20% glucose-PBS solution for 24 hours at +4°C. To enhance the green fluorescence protein (GFP) signal and to facilitate the identification of the MRR, immunofluorescent staining was carried out on 50-μm-thick coronal sections (prepared on a Vibratome VT1200S, Leica, Wetzlar, Germany). Primary antibodies were diluted in Tris-buffered saline (TBS) (Rabbit-anti-Serotonin, 1:10000, ImmunoStar, Hudson, WI, USA; CatNo: 20080; Chicken-anti-GFP, 1:2000, Life Technologies, Carlsbad, CA, USA; CatNo: A10262) and were incubated for 2 days. After washing, sections were incubated in secondary antibody solution overnight (Cy3-conjugated Donkey-anti-Rabbit, 1:500, Jackson ImmunoResearch West Grove, PA, USA; CodeNo:711-165-152; Alexa488-conjugated Goat-anti-Chicken, 1:1000, Life Technologies, CatNo: A-11039; diluted in TBS). After multiple washes, sections were mounted and were evaluated with a Zeiss Axioplan microscope, and images were taken with an Olympus DP70 camera.

The position of the tip of the optical fiber and the size of the virus infected area were determined on micrographs by using on overlay of the stereotaxic atlas images on the series of images of the MRR [17]. We estimated the laser-illuminated volume based on the measurements by Yizhar et al. [18]. Animals were considered to be fully stimulated, when the tip of the optic fiber was located on the dorso-central part of the MRR and the cells of the whole MRR were expressing ChR2 (central stimulation). Cases were considered to be “partial stimulations”, when the whole MRR expressed ChR2, but the tip of the optic fiber was located in the vertical middle third of the MRR or targeted the rostral or caudal one-third of the MRR or illuminated only the paramedian volume of the MRR. Subjects belonged to the “location control” category, when ChR2 was expressed, but the laser illuminated areas outside the MRR. Such areas included the DRN (4 cases), the reticulotegmental nucleus of the pons (2 cases), the pontine reticular nucleus, oral part (1 case), and the paratrochlear nucleus (1 case). “Stimulation controls” were either animals with ChR2 expression in the whole MRR with perfect fiber position, without any illumination, or full illumination in the MRR without ChR2 expression. Mice with week virus expression in the MRR were excluded from the analysis (about 30% of all mice). This failure ratio may seem high, but for unknown reasons the virus was not expressed sufficiently strongly despite the fact that it was injected as with other subjects. Behavior was evaluated in these mice too, as behaviors were scored blind to treatment conditions. In most cases, findings were similar to those observed in mice with full virus expression. Nevertheless, they were discarded to avoid the contamination of the results with insecure information.

### c-Fos immunohistochemistry

90 minutes after photo-stimulation mice were transcardially perfused as above. Frozen coronal sections (30 μm thick) were cut by sliding microtome. Every 6th floating sections were incubated in PBS containing 0.5% Triton X-100 (Calbiochem) and 0.5% H_2_O_2_ (Sigma-Aldrich) for 30 minutes. Non-specific antigens were blocked by 2% bovine serum albumin (BSA; Sigma–Aldrich) in PBS for 30 min at room temperature. The anti-c-Fos primary antibody (rabbit anti-c-Fos, 1:5000, Santa Cruz Biotechnology) was diluted in PBS and sections were incubated for 72 hours at +4°C. After thorough PBS washing sections were incubated for 1h in biotinylated anti-rabbit IgG secondary anti-body (1:500, Jackson ImmunoResearch). Next, sections were incubated in avidin–biotin complex (1:1000, ABC Vectastain Elite kit, Vector Laboratories, Burlingame, CA, USA) diluted in 0.05 M Tris buffered saline (TBS, pH7.6) for 1h at room temperature. After multiple TBS washes, immuno-positive cells were visualized by nickel enhanced 3,3’-diaminobenzidine (DAB, Sigma-Aldrich, St. Louis, MO, USA). Sections were incubated for equal time in Tris-buffered solution containing 0.2 mg/ml DAB, 0.1% nickel-ammonium-sulphate and 0.003% H_2_O_2_. Enzymatic reaction was stopped by TBS washing. Sections were mounted on glass slides in chrome-gelatin solution [0.5% (w/v) gelatin (Sigma–Aldrich) and 1 mM Chromium(III) potassium sulfate dodecahydrate (Sigma–Aldrich)], dehydrated by mixtures of xylol isomers and covered by DPX mounting medium (Sigma–Aldrich). For quantifying c-Fos activation, bright field microscopic images on Olympus BX51 microscope were digitized by an OLYMPUS CCD camera using a 20x magnification lens, and stained particles were counted by means of the Scion Image software (version: 4.0.2; freeware software). The methods of quantification of immune-positive cells see [19].

### Behavioral analysis

#### Behaviors shown during conditioning and test trials

Videotaped trials were analyzed by experimenters blind to treatment conditions by means of a computer-based event recorder (H77, Budapest, Hungary). The following behaviors were all scored and variables were expressed as percent of time spent with the given behavior per total time of the observation. *Ambulation* was assessed by counting line crossings (with all four legs) of a 3x3 grid that divided the cage into nine 10x10 cm squares. Lines were drawn on the video screen, that is, these were not visible to the mice during trials. *Exploration* covered sniffing movements directed towards the floor and walls of the test cage as well as sniffing in the air. *Shock and stimulation runs* were defined by rapid ambulation without engagement in other behaviors that covered at least one cage-length. *Freezing* is complete immobility, no movements of the snout. For representative pictures of locomotion automated video tracking software was used (Noldus EthoVision 10.1, Noldus Information Technology, Wageningen, Netherlands).

The result of the 5 min test was further divided into 10 sec (ON response) and 20 sec (OFF response, averaged to 10 sec for better comparison) time bins in accordance with the optogenetical stimulation during the 50 Hz theta burst protocol (control and continuous 20 Hz stimulation group was divided the same way, regardless of their nature of stimulation). *ON-OFF responses* represent behavioral changes induced by stimulation or shock as compared to behavioral changes observed when the stimulation or shock was stopped. These responses were calculated as follows:

$$ON\;response=\frac{\sum S^{i}-I^{i-1}}{ns}$$

$$OFF\;response=\frac{\sum I^{i}-S^{i-1}}{ni}$$

where *S* is the behavioral score (% time exploration or number of line crossings) recorded during stimulation or shock phases; *I* is the behavioral score recoded during inter-stimulation or inter-shock intervals, *i* is the sequential number of stimulation/shock phases or inter-stimulation/inter-shock intervals, whereas n_S_ or ni is the number of stimulation/shock periods and stimulation/shock intervals, respectively.

We also recorded *central area ambulation* (locomotion within the 4 central squares of the grid overlaid the test cage); *escape jumps* (rapid jumps toward the wall); *grooming* (washing with forepaws and scratching with hindpaws), and *resting* (no locomotion, small postural changes allowed). None of these behaviors were affected at any time-point of the study; therefore, data were not shown.

*Pain sensitivity* was studied on a hot plate (IITC Life Science, Woodland Hills, CA, USA) as described earlier [20]. Pain threshold was expressed as °C, i.e. the temperature at which the animal showed the first sign of nocifensive behavior; the average of three responses was considered the thermal nociceptive threshold [21].

### Experimental design

#### Experiment 1

This study investigated the efficacy of optic stimulation by measuring **serotonin** (5-HT) release *in vitro*. We did not assume that serotonin alone was responsible for the behavioral effects observed in subsequent experiments, as the MRR contains other types of neurons as well [6, 7]. This study was performed only to verify whether the MRR was responsive to photostimulation. ChR2 containing AAV was injected in mice as described above. Eight weeks later mice were anaesthetized; their brains were sampled, sectioned, and processed for 5-HT release. 300-μm coronal brain slices were incubated for 60 min at 37°C in 1 ml Krebs solution containing [^3^H]5-HT (5 μCi/ml). After loading, the tissue was transferred to low-volume superfusion chambers and superfused with 37°C Krebs solution for 60 min by using a peristaltic pump. The Krebs solution was saturated with 95% O_2_ and 5% CO_2_ and temperature was maintained at 37°C throughout. After washing, 1 min perfusate samples were collected and assayed for [^3^H]5-HT by a Wallac 1409 liquid scintillation counter (PerkinElmer, USA). Optical stimulation with 473 nm light (20 mW net energy) started 3 min after the start of sampling. Light was administered continuously for 10 min at 10 (n = 4), 20 (n = 4), 50 (n = 5), and 100Hz (n = 4) frequency. The fiber tip was submerged into the solution and placed to approx. 1 mm above the slice surface. Fractional release was determined by tritium efflux expressed as a percentage of the amount of radioactivity in the slices at the end of the collection period.

To ensure the comparability of findings all behavioral experiments (except Exp.5) were performed in Plexiglas cages (30×30×30 cm) equipped with metallic grid floors. Electric shocks were delivered only in conventional conditioning studies [22]; in stimulation studies electric shocks were replaced by optical stimulation. All trials were performed in the early hours of the light period in a separate, quiet room under normal laboratory illumination (400 lux), and lasted 5 min. Behavior was recorded throughout.

#### Experiment 2

Here we investigated the acute efficacy of MRR stimulation in freely moving mice by studying behavior and c-Fos expression. Mice prepared as described above were transferred to conditioning cages and were stimulated with 473 nm light delivered at 50Hz theta burst frequency (reasons are described later). The following groups were studied: (1) home cage controls (n = 7); (2) no ChR2 controls (transferred for 5 min to the conditioning cage, no ChR2 expression) (n = 7); (3) no light controls (cage transfer, robust ChR2 expression, not stimulated) (n = 7); (4) stimulated by 50Hz theta bursts (cage transfer, robust ChR2 expression, tip of the optical fiber located on the dorso-central part of the MRR (“central”), light administered) (n = 7). Mice were returned to their home-cages after 5 min. 90 min after the beginning of the conditioning trial mice were euthanized and their brains were processed as described above.

#### Experiment 3

**Fig 1:**
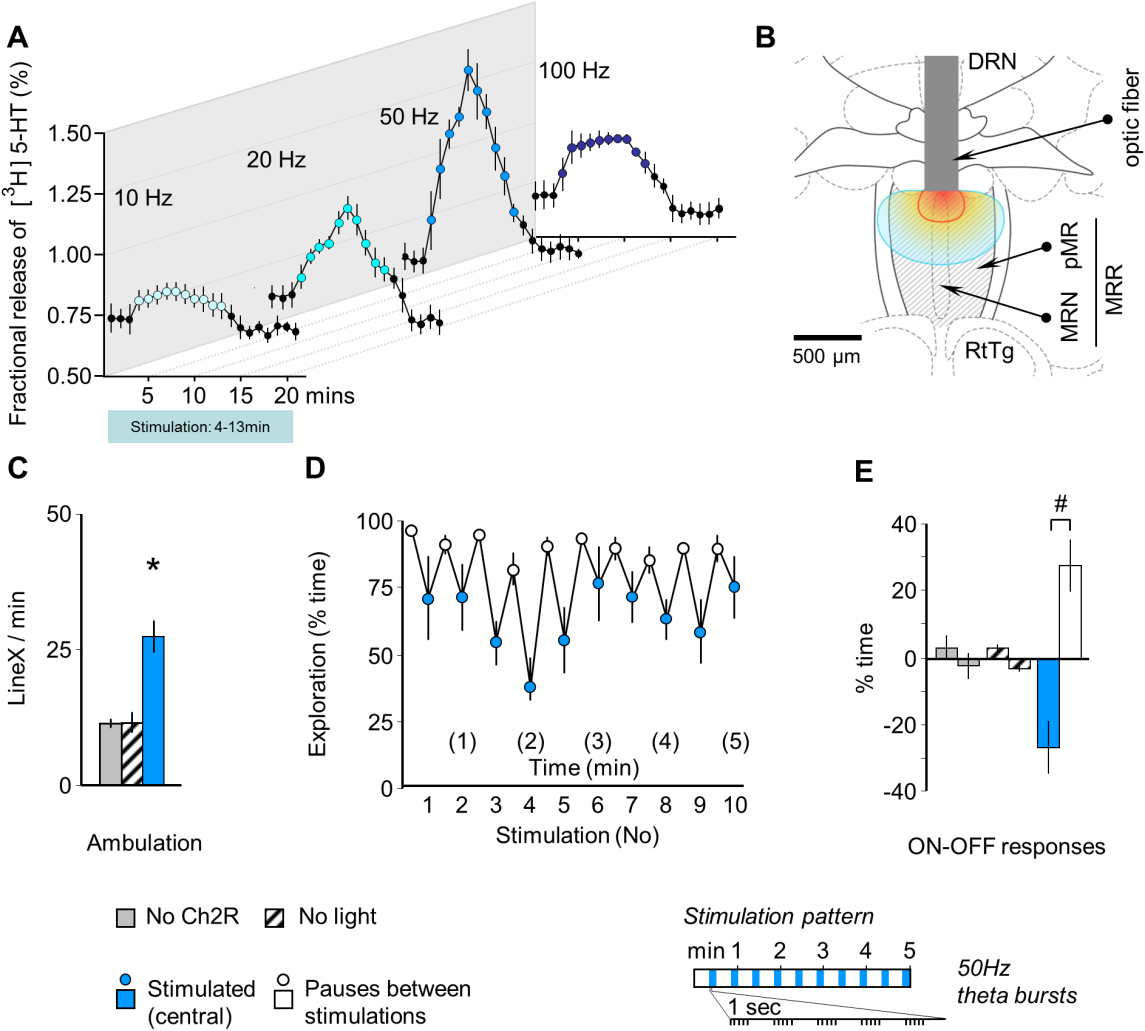
Channelrhodopsin (ChR2)-mediated optic stimulation robustly activated the MRR and altered behavior. **(A**) Effects of *in vitro* stimulation on ^3H^5-HT release from coronal brain slices including the MRR, which demonstrates the responsiveness of the area to photostimulation. The time-resolution of the curves is 1 min and covers the 10 min stimulation periods indicated by color. (**B**) The location of optic fibers in the *in-vivo* experiments, which are presented in panels C-E. All mice showed robust ChR2 expression in the MRR. Red and blue lines show iso-intensity lines of light penetration at 10% and 1% of release intensity, respectively (based on [18]). (**C**) MRR stimulation increased ambulation in freely moving animals. Mice were connected to the stimulation equipment by optic fibers, were transferred into a novel cage to mimic the conditions of the subsequent MRR-conditioning study and were stimulated at 50Hz theta-burst frequency by blue light (“stimulated (central)”). Controls were either stimulated in the absence of ChR2 expression (“no ChR2”), or ChR2 expression was induced, but light was not administered (“no light”). (**D**) Rhythmic decrease in exploration was induced by intermittent (rhythmic) stimulation of the MRR. (**E**) Rhythmic changes illustrated as *ON-OFF responses*, i.e. changes in behavior elicited by the onset of stimulations *(ON responses)* and those elicited by their halting *(OFF responses)*. *DRN*: dorsal raphe; *MRN*: median raphe; *MRR*: median raphe region; *LineX*: line crossings; *pMR*: paramedian raphe; *RtTg*: reticulotegmental nucleus of the pons. * p<0.01 significant difference from “no ChR2” and from “no light”; # p<0.01 significant *ON-OFF* difference.

Acute (during stimulation), recent (1 day) and remote (7 days) [23] consequences of ChR2-mediated MRR stimulation was studied in comparison with those of electric shocks. This study was performed in a different set of animals that were naive to experimentation. Optic stimulation patterns for MRR-conditioning were selected based on discharge frequencies recorded in MRR neurons [24] and were also influenced by the findings of *in vitro* studies, where continuous 20Hz stimulation maintained 5-HT release for at least 6 min, whereas release declined within 5min with continuous 50Hz stimulation (**Fig 1A**). Note that the length of MRR conditioning trial was 5min. Also noteworthy is that continuous 50Hz discharges were not recorded in the MRR, but bursts of 50Hz emerging at theta frequency were observed [24]. Consequently, mice were either stimulated continuously at 20Hz or intermittently at 50Hz theta-burst frequency. In the latter protocol, 10 sec-long stimulations were delivered every 30 sec, i.e. each mouse received 10 stimulations over 5 min. Stimulations were separated by 20 sec pauses. Within stimulation phases, 5 stimulation bursts of 50 Hz were delivered per sec. No mouse received electric shocks in these groups. During test trials mice were exposed to the cage associated with MRR stimulation 1 and 7 days after stimulation (i.e. on day 2 and day 8 of the experiment). To ensure full contextual similarity, they were connected to the stimulation equipment by optic fibers, but no stimulation was delivered.

During conventional fear conditioning trials, half of the intact mice were exposed to electric shocks in the same boxes where MR-conditioning was performed. Shocks were administered every 30 sec (i.e. each subject received 10 shocks during 5 min) in the form of shock trains that lasted 1 sec at 50 Hz. Conditioned fear was tested 1 and 7 days after conditioning. No shocks were administered this time.

In this paradigm, the following groups were studied (1) intact mice without electric footshock (n = 7); (2) intact mice with electric footshock (n = 9); (3) stimulation control (n = 12); (4) location control stimulated at 50Hz theta burst frequency (n = 6); (5) mice stimulated at 50Hz theta burst frequency (partial: n = 6; central: n = 12); (6) mice stimulated at continuous 20 Hz (partial: n = 8; central: n = 9). The optogenetically stimulated groups (5 and 6) were further subdivided based on the location of the tip of optic fibers which is indicated in parentheses.

#### Experiment 4

The effects of MRR silencing was studied on behavioral responses elicited by conventional footshock conditioning. All mice were prepared as shown above except that the virus carried the NpHR gene this time, and continuous MRR stimulation was performed with 593 nm (yellow) laser beam. All mice showed robust NpHR expression and optic fibers were located in the dorso-central position of the MRR. During the conditioning trial all mice were exposed to electric shocks as described above [22]. Shocks were associated with MRR silencing by yellow light in half of the mice, whereas the other half received shocks without MRR illumination. Illumination was applied during the 5 min footshock and ended 25 min later, i.e. lasted altogether for 30 min. In test trials mice were connected to optic fibers to ensure full contextual similarity, but neither shocks nor illumination was administered. Mice were tested 1 and 7 days after conditioning. The following groups were compared (1) shock control (robust NpHR expression no shock and no light administered; n = 7); (2) shocked (robust NpHR, received electric shocks, no light administered; n = 10); (3) inhibited (robust NpHR expression, received electric shocks, MRR inhibited by yellow light during training; n = 14).

#### Experiment 5

The effects of MRR silencing on pain perception was studied. Mice were prepared as described for *Experiment 4* and yellow light was administered as shown above. The hot-plate test was performed in the same animals on two consecutive days in the randomized presence or absence of MRR silencing. Each trial started by a 10 min habituation period. Three trials were run each day; the inter-trial interval was 1 min. Pain threshold was averaged over trials.

### Statistics

Data were analyzed using STATISTICA 12.0 software package (StatSoft, Inc., Tulsa, OK, USA). Data were shown as means ± standard error of the mean. Main effects were investigated by one- (factor ‘group’) or two-way (factors ‘group’ and ‘response *(ON-OFF response)*’) analysis of variance (ANOVA) or with repeated measures ANOVA (factors ‘group’; repeated factor ‘time’). Significant main effects where further analyzed by the Duncan post-hoc test. Multiple regression method was used to analyze the correlation between behavior and c-Fos activity. P< 0.05 was considered significant.

## Results

### Optic stimulation activated the MRR

The illumination of the ChR2-containing MRR led to frequency-dependent release of 5-HT *in vitro* (**Fig 1A**). This release gradually decreased after about 5–7 min constant stimulation likely because of the exhaustion of neuronal capacities; as such, stimulations were restricted to 5 min in all subsequent experiments. The highest increase was elicited by 50Hz stimulation, while illumination by blue light at 20Hz resulted in a similarly remarkable 5-HT release. Therefore these two frequencies were used in the following. This study demonstrated that the MRR was responsive to photostimulation.

In freely moving mice, intermittent optic stimulation of the dorso-central area of the MRR (**Fig 1B**) at a 50 Hz theta-burst frequency increased locomotion (F_(2,18)_ = 19.42, p< 0.01) (**Fig 1C**) and entrained a noticeable behavioral rhythm that was characterized by reduced exploration during the administration of stimuli (*ON-OFF response*: F_(1,18)_ = 6.00, p< 0.05; group x response interaction: F_(2,18)_ = 11.76, p< 0.01), (**Fig 1D and 1E**).

**Fig 2:**
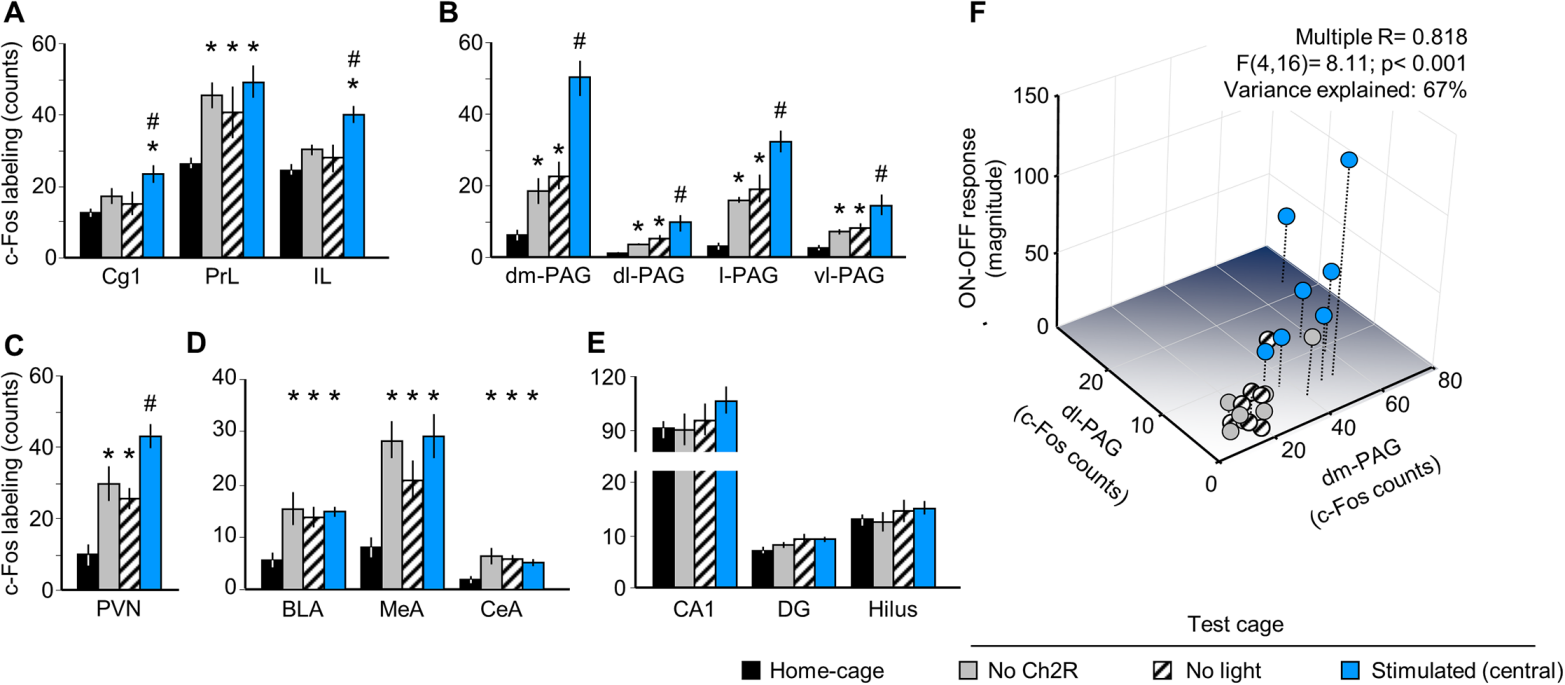
MRR stimulation selectively activates brain areas involved in emotional control. Optic stimulation selectively increased the expression of the activity marker c-Fos in two sub-regions of the medial prefrontal cortex (**A**), the whole periaqueductal gray (**B**), and the paraventricular nucleus of the hypothalamus (**C**). PrL and the amygdala was activated by cage transfer, but not stimulation (**D**); the hippocampus showed no responses (**E**). Panel **F** is a 3D illustration of the Multiple Regression analysis presented in the text. *BLA*: basolateral amygdala; *CA1*: CA1 region of the hippocampus; *CeA*: central amygdala; *Cg1*: anterior cingulate cortex; *DG*: dentate girus of the hippocampus; *dl-*: dorsolateral; *dm-*: dorsomedial; *IL*: infralimbic cortex, *l-*: lateral; *MeA*: medial amygdala; *PAG*: periaqueductal gray; *PrL*: prelimbic cortex; *PVN*: paraventricular nucleus of the hypothalamus; *vl-*: ventrolateral. * p<0.05 significant difference from home-cage controls; # p<0.05 significant difference from “no ChR2”.

We also investigated in the same mice the expression of the neuronal activity marker, c-Fos in brain areas relevant to emotional control (**Fig 2**). There was significant difference between groups in the three main subdivision of the medial prefrontal cortex (mPFC), namely in the anterior cingulate (Cg1) (F_(3,24)_ = 3.56, p<0.05), infralimbic (IL) (F_(3.24)_ = 6.72, p<0.01) and prelimbic cortices (PrL) (F_(3.24)_ = 4.37, p<0.05). Moreover, a group effect was also significant in periaqueductal gray (PAG) subdivisions (**S4 Fig**), namely in the dorsomedial (F_(3.24)_ = 25.55, p<0.01), dorsolateral (F_(3.24)_ = 7.19, p<0.01), lateral (F_(3.24)_ = 21.94, p<0.01) and ventrolateral (F_(3.24)_ = 7.98, p<0.01) PAG, as well as in the paraventricular nucleus of the hypothalamus (PVN) (F_(3.24)_ = 9.95, p<0.01) and in basolateral (F_(3.24)_ = 4.86, p<0.01), medial (F_(3.24)_ = 8.19, p<0.01), central (F_(3.24)_ = 3.97, p<0.05) amygdala, but not in hippocampus. Post hoc analysis showed that MRR stimulation significantly increased c-Fos counts in Cg1 and IL, but not in PrL, all sub-regions of PAG, as well as in PVN (**Fig 2A–2C**). Neither the amygdala nor the hippocampus responded specifically to optic stimulation (**Fig 2D and 2E**).

A Multiple Regression Analysis showed that PAG activation ‒ with a significant individual contribution of the dorsomedial (β = 1.07, p<0.001) and dorsolateral PAG (β = -0.76, p<0.01) ‒ explained a large share of variation in behavioral responses including both the magnitude of *ON-OFF responses* (F_(4,16)_ = 8.11; p< 0.001; β = 0.82; Variance explained (R^2^): 67%) and the increase in ambulation (F_(4,16)_ = 3.02; p< 0.05; β = 0.66; Variance explained (R^2^): 43%) (**Fig 2F**). Prediction power was not increased meaningfully when the Cg1 and IL were added to the model (Variance explained (R^2^): 69–70%).

### MRR conditioning induces agitation but not fear in the conditioning trial

**Fig 3:**
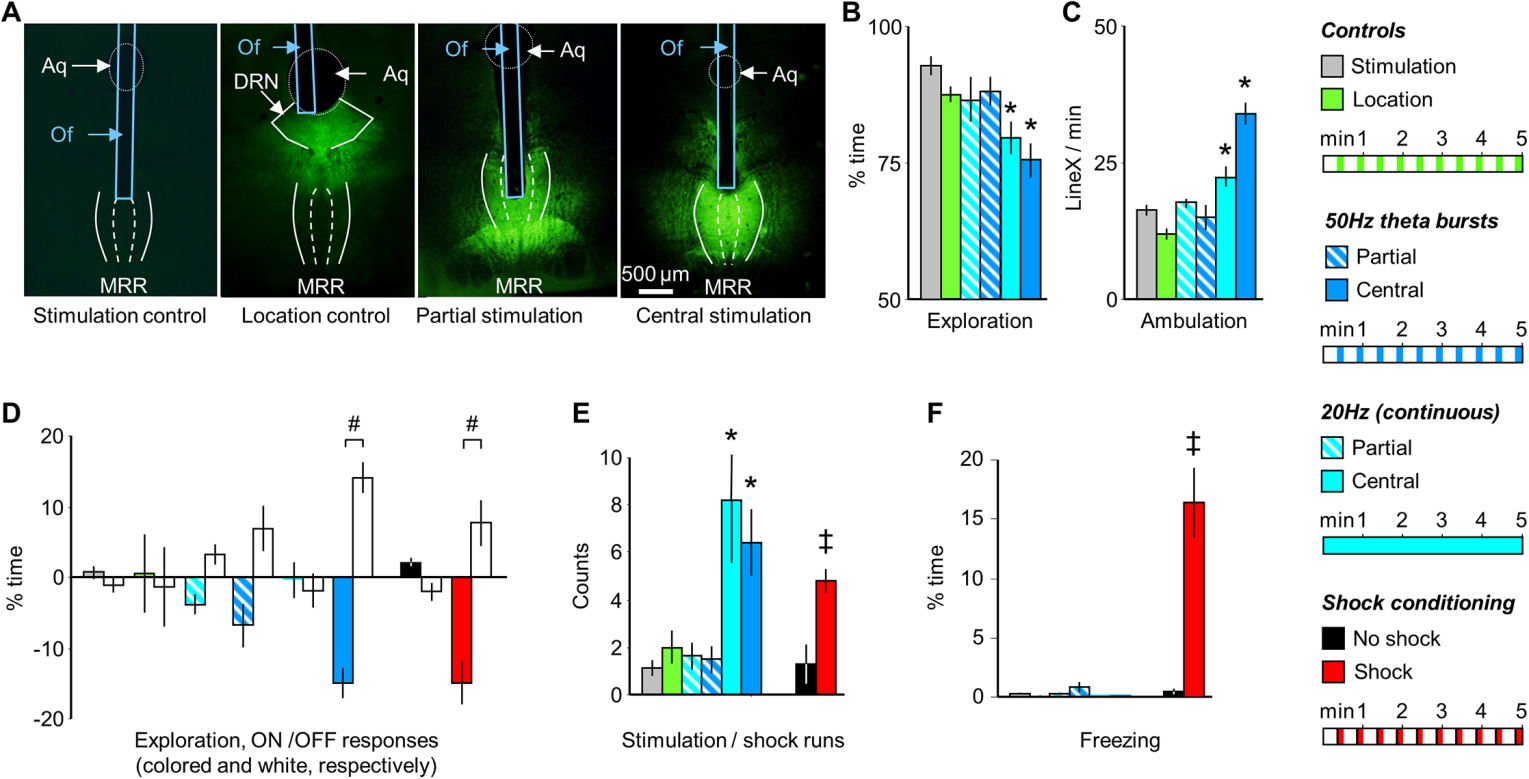
The acute effects of MRR- and shock-conditioning are differentiated by freezing. **(A**) Photomicrographs illustrating the location of the tip of the optic fibers and the distribution of GFP-labeled ChR2 expression. For stimulation patterns see the right-hand side of the figure. (**B**) and (**C**) MRR stimulation decreased exploration and increased ambulation only when it targeted the dorso-central region of the MRR ("central stimulation"). Partial stimulations (that reached ventral, lateral, anterior or posterior aspects of the MRR) were ineffective. (**D**) The rhythmic delivery of 50Hz theta bursts induced a corresponding rhythm of behavioral changes as indicated here by *ON-OFF responses*. Actual behavioral rhythms were similar to which is shown in Fig 1D and were presented in S1 Fig. Note that behavior scoring was time-structured in a similar fashion in all groups to allow their comparison. (**E**) Central, but not partial MRR stimulations elicited “runs”, which were behaviorally similar to those observed in shocked mice. (**F**) Freezing was readily elicited by shock administration, but not by MRR-conditioning. *Aq*: aqueductus cerebri; *DRN*: dorsal raphe nucleus; *MRR*: median raphe region; *LineX*: line crossings; *Of*: optic fiber; *ON-OFF responses*: average changes in behavior elicited by the onset of stimulation *(ON responses)* and those elicited by their halting *(OFF responses)*; *stimulation/shock runs*: episodes of rapid ambulation without exploration. * p<0.01 significant difference from stimulation controls (either light-stimulated without ChR2 expression, or ChR2 expression without stimulation); # p<0.01 significant *ON-OFF* differences; ‡ p<0.01 significant difference from shock controls.

Stimulations that targeted the dorsal aspects of the MRR and at the same time were located centrally along the rostrocaudal and lateral extensions of the region ("central stimulation", **Fig 3A**) resulted in an overall decrease in exploration (F_(5,52)_ = 6.94, p<0.01) and increase in ambulation (F_(5,52)_ = 27.13, p<0.01) with both (20Hz and theta burst) stimulation protocols (**Fig 3B and 3C**). Moreover, when administered rhythmically at 50Hz theta burst frequency it entrained the behavioral rhythm with marked difference in *ON-OFF responses* of exploration seen in the previous study (*ON-OFF response*: group: F_(5,52)_ = 2.89; p<0.05; response: F_(1, 52)_ = 9.81; p<0.01; interaction: F_(5,52)_ = 5.23, p<0.01), (**Fig 3D;** similarly to **Fig 1D and 1E;** for ambulation see: **S1 Fig**). In addition, both stimulation paradigms induced "stimulation runs" (F_(5,52)_ = 5.62, p<0.01), which were displayed at similar frequencies (**Fig 3E**). Decreased exploration and increased stimulation runs of MRR stimulated mice were represented by higher velocity (for representative pictures see: **S3A** and **S3B Fig**: more yellow color) and less time spent in a given area (for representative pictures see: **S3C** and **S3D Fig**: less red color) compared to non stimulated controls (**S1 Video)**.

Electric shocks, when administered intermittently, also entrained rhythmic changes in exploration (*ON-OFF responses*: *exploration*: group: F_(1,14)_ = 13.45, p<0.01; response: F_(1,14)_ = 5.10, p<0.05; interaction: F_(1,14)_ = 13.29, p<0.01), (**Fig 3D**) and elicited runs (F_(1,14)_ = 18.20, p<0.01), (**Fig 3E**). However, shocks also elicited fear as shown by a marked increase in freezing behavior (F_(1,14)_ = 24.92; p<0.01), (**Fig 3F**). Freezing was found to be a common response to electric shocks in mice, was displayed during the pauses that separated shocks, and increased over the course of conditioning [25]. In contrast to shock-conditioning, freezing was absent during the fear conditioning session in mice submitted to MRR-conditioning (**Fig 3F**).

The acute behavioral effects of MRR stimulation were absent in controls i.e. in mice where ChR2 was expressed, but light stimulation was not administered and in mice stimulated with light in the absence of ChR2 expression ("stimulation controls"). Neither group differed from intact mice (**S2 Fig**). In addition, post hoc comparison revealed that the behavioral effects of stimulation showed a marked topographical specificity as no behavioral changes were induced by stimulations that targeted ventral, rostral, lateral, or caudal regions of the MRR ("partial stimulation") or by stimulations targeting areas neighboring the MRR ("location controls") (**Fig 3A–3F**).

### MRR stimulation led to a late-onset conditioned fear

**Fig 4:**
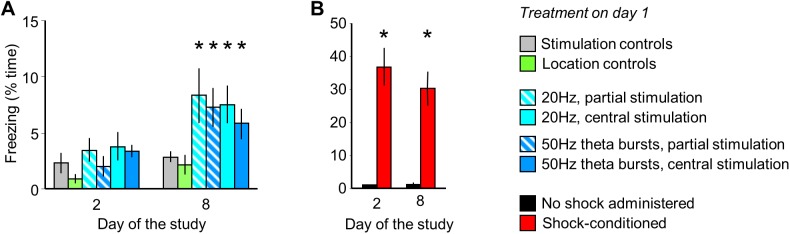
MRR stimulation led to the late-onset development of conditioned fear. **A**. Freezing by MRR-conditioned mice one and seven days after MRR-conditioning (day 2 and 8 of the study). The MRR was stimulated on day 1. **B**. Freezing by intact mice submitted to shock-conditioning on day 2 and 8. * p<0.05 significant difference from stimulation or location/no-shock controls.

Although MRR stimulation did not elicit fear during the conditioning trial (**Fig 3F**) and the acute effects of stimulation were reversible on a very short time-scale (**Figs 1D, 3D and S1 Fig**), the re-exposure of previously stimulated mice to the conditioning cage without any further stimulation led to slow development of contextual fear. No behavioral alterations were observed the next day, but a freezing response developed over 7 days (group: F_(5,52)_ = 2.94; p<0.05; time: F_(2,104)_ = 42.94, p<0.01; interaction: F_(10,104)_ = 2.06, p<0.05), (**Fig 4A**). By contrast, electric shock-conditioned mice displayed conditioned fear on both test days (group: F_(1,14)_ = 27.77, p<0.01), (**Fig 4B**). The stimulation-dependence and MRR-specificity of delayed effects was ensured as no changes in freezing were observed in stimulation and location controls (**Fig 4A; S2 Fig**).

### MRR silencing during electric shock-induced fear conditioning: Acute and remote effects

**Fig 5:**
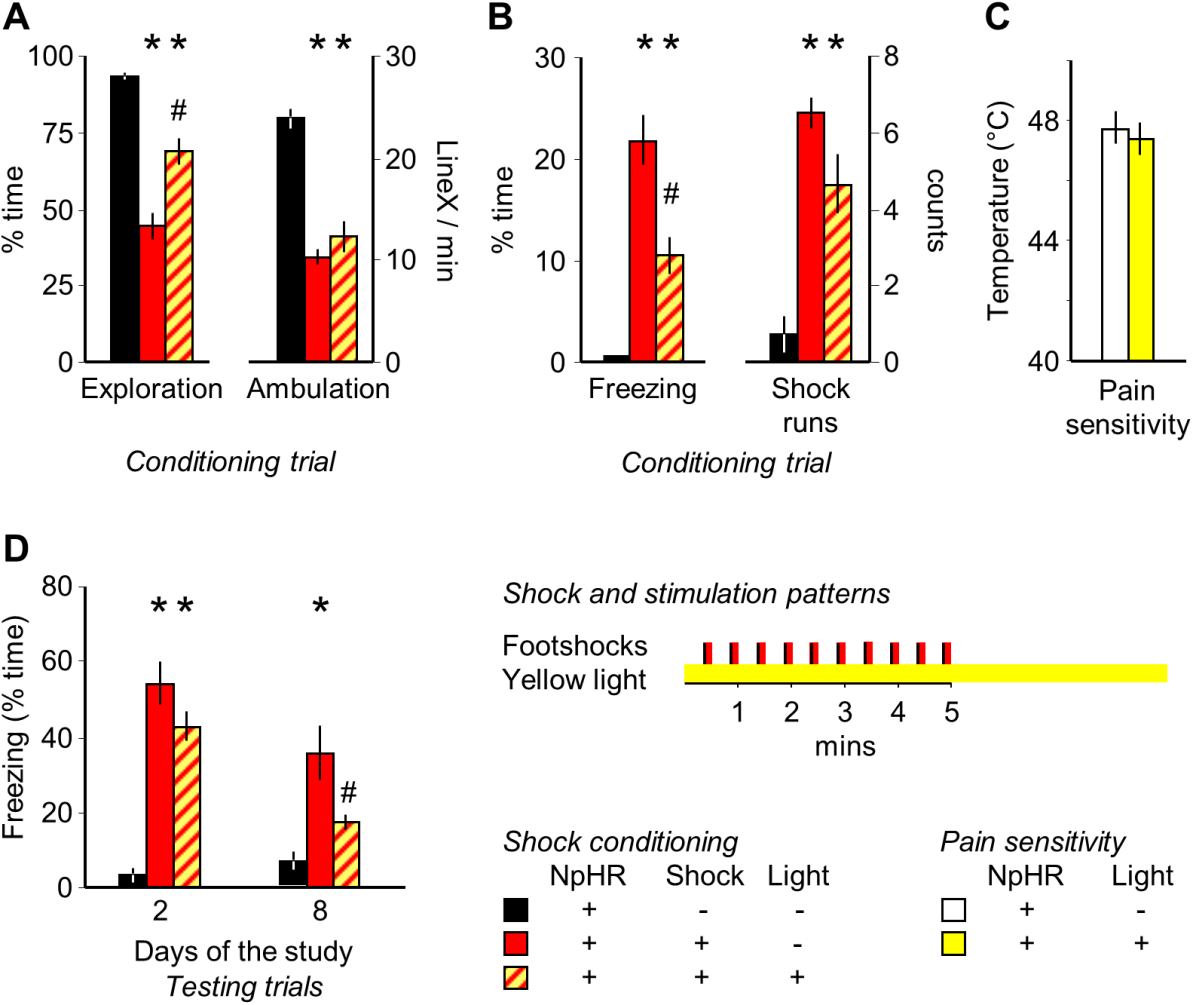
Halorhodopsin-mediated silencing of the MRR ameliorates acute and remote, but not recent effects. Mice were submitted to electric shock conditioning either in a regular way or while their MRR was silenced by halorhodopsin illumination by yellow light. Halorhodopsin was expressed in all mice by a viral vector that carried the NpHR gene, and all mice were connected to optic fibers. (**A)** and (**B**) The acute effects of electric shocks were partially ameliorated by MRR silencing during the conditioning trial. (**C**) Effects were not secondary to alterations in pain perception, which was studied in the hot-plate and expressed as the temperature that consistently elicited paw licking. (**D**) Halorhodopsin silencing did not affect recent conditioned fear 24h after shock-conditioning, but markedly ameliorated remote freezing 7 days later. Note that freezing was statistically similar in MRR-silenced and non-shocked groups. * p<0.05 significant difference from non-shocked; # p<0.05 significant difference from shocked.

To crosscheck for the effects of optical stimulation, we exposed subjects to electric footshocks while their MRR was silenced. This procedure had a significant impact on the behavioral effects of electric shocks (exploration: F_(2,29)_ = 28.30, p<0.01, ambulation: F_(2,29)_ = 22.31, p<0.01, freezing: F_(2,29)_ = 23.37, p<0.01 and shock runs: F_(2,29)_ = 13.44, p<0.01). Post hoc analysis showed, that shock exposure decreased exploration and ambulation in the training trial, and elicited freezing and shock runs in the same period (**Fig 5A and 5B**) and some of these effects, particularly the decrease in exploration and the increase in freezing were dampened by concurrent MRR silencing. The effect was not secondary to alterations in pain sensitivity, as this was not altered by MRR inhibition (**Fig 5C**). Silencing did not affect fear responses the next day, but fear responses were significantly reduced 7 days later (group: F_(2,29)_ = 22.02, p<0.01; time: F_(1,29)_ = 29.76, p<0.01; interaction: F_(2,29)_ = 9.42, p<0.01); practically to the non-shocked level (no significant difference between non-shocked and MRR silenced-shocked groups), (**Fig 5D**).

## Discussion

The optogenetic activation of the MRR did not trigger freezing acutely, demonstrating that this brain region detaches from fear-effector systems, which elicit freezing when stimulated [1, 2]. Yet MRR stimulation induced agitation that likely reflected its unpleasantness as shown by reduced exploration and shock-runs. Despite the agitation produced, MRR activation did not evoke conditioned fear responses the next day, which indicates that MRR stimulation alone was not sufficient to form an associative memory that linked an adverse experience to a context. By contrast, MRR activation transformed a likely adverse, but not fearful experience into a long-term fear memory trace after a week. It has been demonstrated earlier that MRR has a large impact on the circuits that process stressful experiences, encode them as fear memories, and retrieve this information when the subject is re-exposed to contexts or cues reminiscent of fearful experiences, i.e. the general belief is that its role is indirect [10–12, 14, 15]. Here we show that in addition to this established role, MRR stimulation may *per se* elicit the emergence of remote fear memories ‒ surprisingly in the absence of recent ones. The main question raised by this finding is how fear emerged over time, if stimulation *per se* was not fearful nor did it elicit fear 24h after conditioning. In brief, how can fear memories develop "retrospectively" i.e. in the absence of fear learning? We propose that this phenomenon resulted from the sensitization of the fear circuitry to the recall of aversive memories.

MRR stimulation resulted in agitation as indicated by the reduction in exploration ‒ a behavior of key importance in novel environments under normal conditions ‒ and by the emergence of stimulation runs, a behavior commonly observed in mice exposed to aversive stimuli e.g. electric shocks [25]. We suggest that agitation produced by MRR stimulation was aversive, because in addition to the emergence of runs, it also resulted in the activation of the PVN, which orchestrates the stress response, of the Cg1, which is involved in the processing of aversive stimuli [26] and of the PAG, which coordinates behavioral responses to aversive situations [27]. Furthermore, MRR efferents were demonstrated to contribute to the encoding of aversive expectations [15]. It occurs, however, that agitation—possibly aversiveness—elicited by MRR stimulation was insufficient to result in fear as demonstrated by the absence of freezing, and by the lack of activation by the amygdala and hippocampus, which are strongly involved in the processing of fear [28, 29], and ‒ as demonstrated by the Multiple Regression analysis ‒ by the relatively low behavioral impact of the ventral PAG, which controls fear-induced freezing [27].

However, the optogenetic stimulation of the central MRR at a 50 Hz theta-burst frequency (a discharge pattern readily observed in MRR neurons [24]), administered at a rate that is commonly used in electric footshock-induced fear paradigms (2 events per min over 5 min) [30]—readily altered behavior e.g. it increased ambulation and reduced exploration. Noteworthy, these behavioral effects of stimulation were reversible on a very short time-scale.

A strong correlation was detected between *ON-OFF responses* (differences between the stimulation and non-stimulation periods) and the activation of the dorsomedial and dorsolateral PAG. These findings suggest that behavioral responses elicited by stimulation were specifically mediated by the PAG, which coordinates behavioral responses to aversive situations in general [31], whereas its dorsal aspects ‒ uniquely associated with stimulation-induced behaviors in this study ‒ are involved in the induction of flight [27], a behavior somewhat similar to the increase in ambulation and decrease in exploration observed in our stimulated mice.

Besides these immediate effects, the MRR and the brain areas that were activated by it e.g. the PAG, mPFC and the PVN may have affected the fear circuitry on the long run. Indeed, plastic neural changes that underlie durable acquired fear are believed to be under the control of serotonergic and glutamatergic mechanisms hosted by the MRR, as well as by the PAG, the mPFC and by the stress response orchestrated by the PVN [4–9, 28, 32, 33]. We propose that when mice were re-exposed to the conditioning cage one week after MRR-conditioning, adverse memories were recalled on the background of a fear circuitry that was sensitized by plastic changes. Fear was observed 7 days after MRR conditioning and it did not depend on the stimulated MRR region (central or partial stimulations). This was in sharp contrast with electric shock conditioning, where fear-like responses were present throughout. Interestingly, the acute behavioral effects of MRR stimulation and the induction of late-onset fear responses were dissociable: the former were triggered by the stimulation of the dorso-central MRR (central stimulation), whereas the latter seemed to be related to MRR stimulation in general (i.e. it was produced by both central and partial stimulations).

Taken together, the considerations presented above suggest that aversive memories were retrospectively transformed into fear memories, because coping with the recalled memory trace was altered. Our hypothesis naturally needs further scrutiny, but irrespective to its validity, our findings reveal a specific role for the MRR in fear learning. The delayed induction of fear by the stimulation of a phylogenetically ancient subcortical center, the MRR, may be perceived as a proof for its involvement in the mechanisms that make fear persistent.

To further confirm the stimulatory role of the MRR in the development of remote fear memories, we exposed subjects to electric footshocks and silenced their MRR by NpHR. The effects of MRR silencing supported the findings of the MRR-conditioning study. During the conditioning trial, MRR stimulation decreased, whereas MRR silencing increased exploration. The two treatments also shared the property of not influencing recent, but having an impact on remote conditioned fear.

Earlier findings showed that fear learning is subserved by interconnected but distinct mechanisms, where fear expression is under the control of fear-effector systems. Here we suggest that the persistent encoding of fear memories is—at least partly—elicited by the MRR. The elucidation of the relationship between these two systems may substantially improve our understanding of the phenomena that transform adverse experience into persistent fear memories, and consequently, the mechanisms that underlie of trauma- and stressor-related disorders.

## Supporting information

S1 Fig Behavioral effects of intermittent 50Hz theta bursts and continuous 20Hz stimulation. **(a**) Time course of exploration and ambulation shown in time-bins representing the stimulation and non-stimulation periods of intermittent stimulation at 50Hz theta burst frequency. (**b**) Exploration and ambulation in mice submitted to continuous 20Hz stimulation in time-bins corresponding to those employed in panel *a*. (**c**) ON-OFF responses for ambulation. See Fig 3 for explanation and for ON-OFF responses in exploration. ^#^, p< 0.05 for ON-OFF differences.
(TIF)

S2 Fig Behavior in control groups was not different from that seen in intact mice. The scale of Y-axes is similar to those used for experimental groups (see Figs 1 and 3). (**a**) No rhythmic changes in exploration or ambulation were observed. Note that the scoring of behavior was time-structured in all groups as for effective intermittent stimulations (as in Fig 1). (**b**) The lack of rhythmic changes in behavior is also shown by the lack of differences between stimulation phases and inter-stimulation intervals *(ON-OFF responses)*. (**c**) The number of "runs" ‒readily elicited by both effective MRR stimulation and electric shocks‒ was low in controls, and was not affected by treatments. (**d**) Freezing remained low in controls throughout the experiment and was not affected by treatments.
(TIF)

S3 Fig Representative pictures of the locomotion on day 1. Track visualization of the distance travelled during the fear conditioning. Higher velocity is indicated by bright color (yellow), slower movements or rest is signed by dark red (**a,b**). Heat maps represent the average time spent (s) at each location (**c,d**), blue meaning less time and red marking the opposite. Track visualization and heat maps show one representative example from the stimulation control *(see* *methods; this is a case of no stimulation)* and another from the central stimulation group *(see* *methods; intermittent stimulation at 50Hz theta burst frequency)*.
(TIF)

S4 Fig Representative pictures of the periaqueductal gray after c-Fos immunohistochemistry. Pictures were taken around -4.36 mm from the Bregma. Scalebar is 100μm. Group1: home cage controls; Group2: no ChR2 controls *(transferred for 5 min to the conditioning cage*, *no ChR2 expression)*; Group3: no light controls *(cage transfer*, *robust ChR2 expression*, *not stimulated)*; Group 4: stimulated by 50Hz theta bursts *(cage transfer*, *robust ChR2 expression*, *tip of the optical fiber located on the dorso-central part of the MRR (“central”)*, light administered). DMPAG: dorsomedial part of periaqueductal gray (PAG); DLPAG: dorsolateral PAG; LPAG: lateral PAG; VLPAG: ventrolateral PAG; DR: dorsal raphe nucleus.
(TIF)

S1 Video Representative video of the locomotion on day 1. Video shows one representative example from the stimulation control *(see* *methods; this is a case of no stimulation)* and another from the central stimulation group *(see* *methods; intermittent stimulation at 50Hz theta burst frequency)*.
(AVI)

S1 File Raw data of the experiment. File contains all data underlying the findings described in the manuscript and grouped by the number of the figures.
(XLS)
