# Supplementary figures and images for: Median raphe region stimulation alone generates remote, but not recent fear memory traces
Source: PLoS One. 2017 Jul 14;12(7):e0181264. doi: 10.1371/journal.pone.0181264 (PMC5510848; doi:10.1371/journal.pone.0181264)

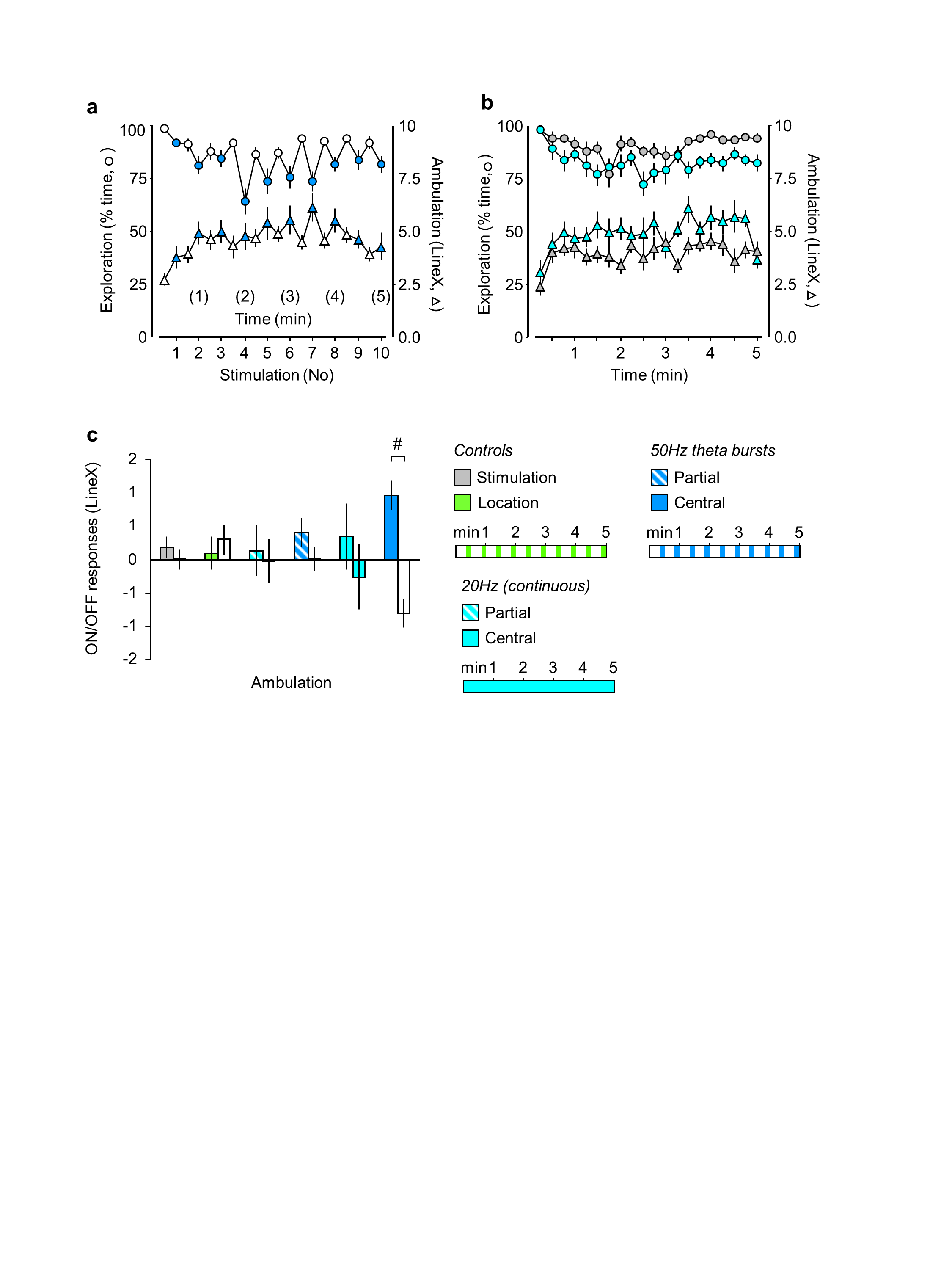

Supplement: S1 Fig — (a) Time course of exploration and ambulation shown in time-bins representing the stimulation and non-stimulation periods of intermittent stimulation at 50Hz theta burst frequency. (b) Exploration and ambulation in mice submitted to continuous 20Hz stimulation in time-bins corresponding to those employed in panel a. (c) ON-OFF responses for ambulation. See Fig 3 for explanation and for ON-OFF responses in exploration. #, p< 0.05 for ON-OFF differences. (TIF) [file pone.0181264.s001.TIF]

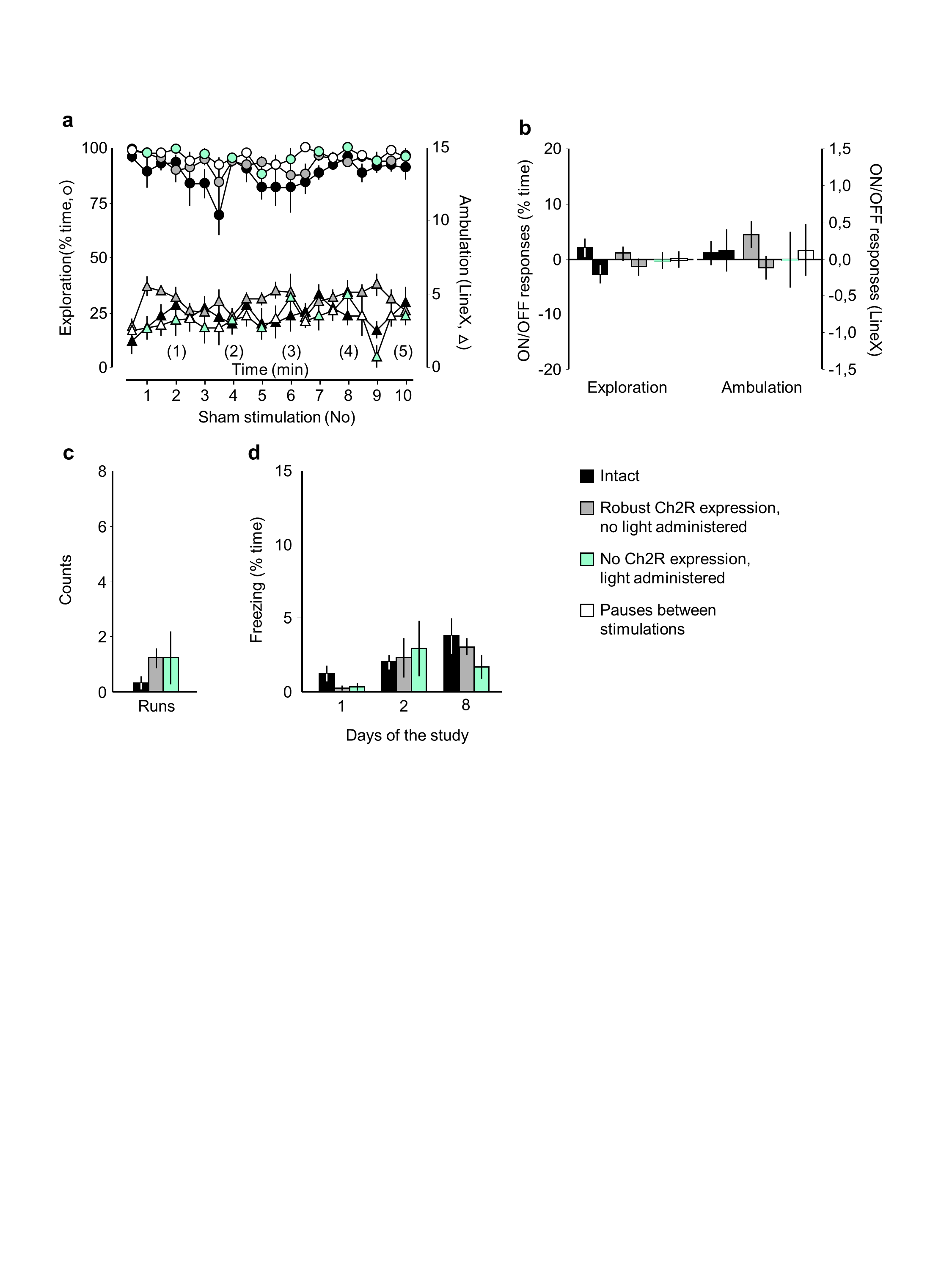

Supplement: S2 Fig — The scale of Y-axes is similar to those used for experimental groups (see Figs 1 and 3). (a) No rhythmic changes in exploration or ambulation were observed. Note that the scoring of behavior was time-structured in all groups as for effective intermittent stimulations (as in Fig 1). (b) The lack of rhythmic changes in behavior is also shown by the lack of differences between stimulation phases and inter-stimulation intervals (ON-OFF responses). (c) The number of "runs" ‒readily elicited by both effective MRR stimulation and electric shocks‒ was low in controls, and was not affected by treatments. (d) Freezing remained low in controls throughout the experiment and was not affected by treatments. (TIF) [file pone.0181264.s002.TIF]

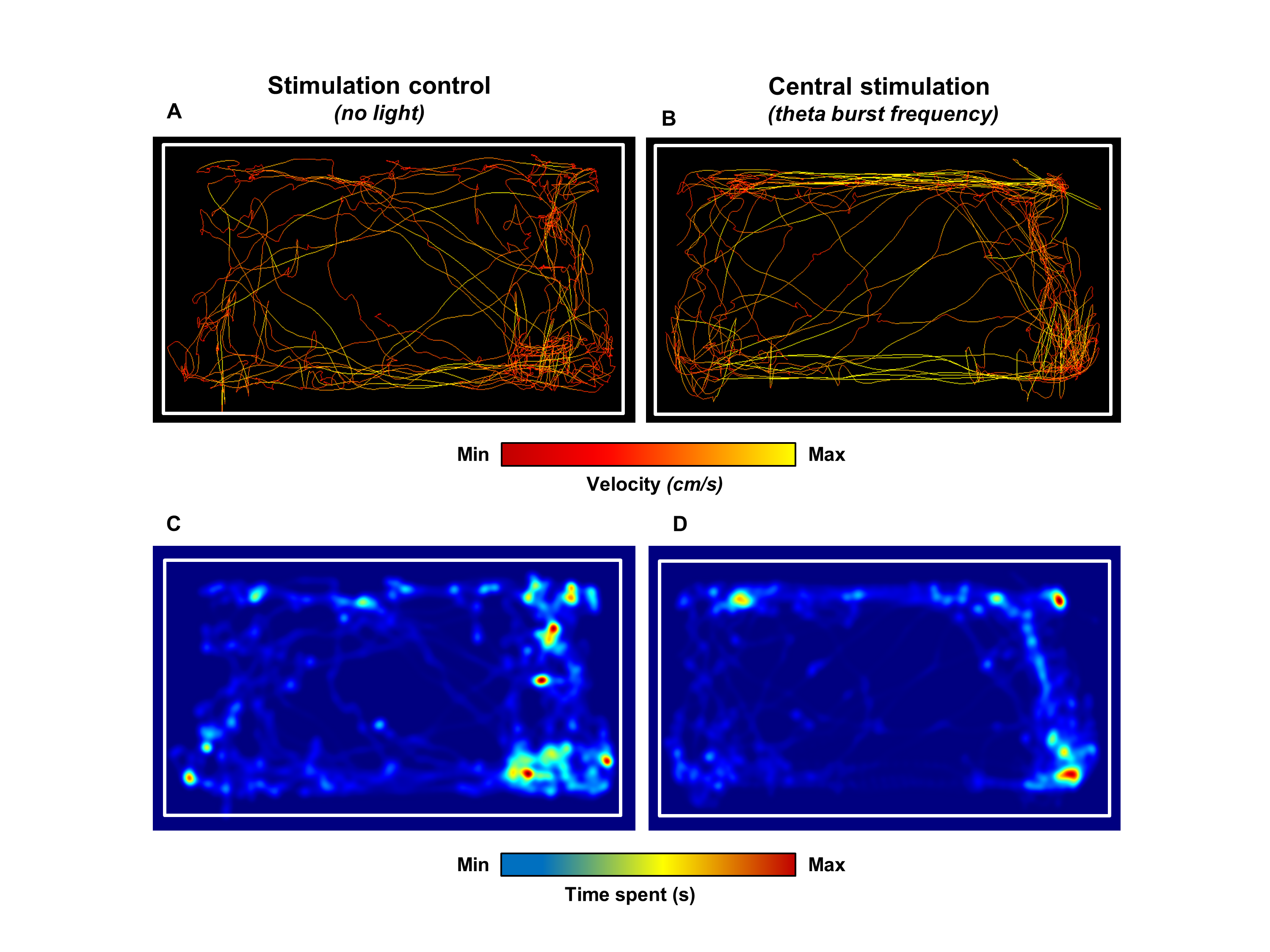

Supplement: S3 Fig — Track visualization of the distance travelled during the fear conditioning. Higher velocity is indicated by bright color (yellow), slower movements or rest is signed by dark red (a,b). Heat maps represent the average time spent (s) at each location (c,d), blue meaning less time and red marking the opposite. Track visualization and heat maps show one representative example from the stimulation control (see methods; this is a case of no stimulation) and another from the central stimulation group (see methods; intermittent stimulation at 50Hz theta burst frequency). (TIF) [file pone.0181264.s003.TIF]

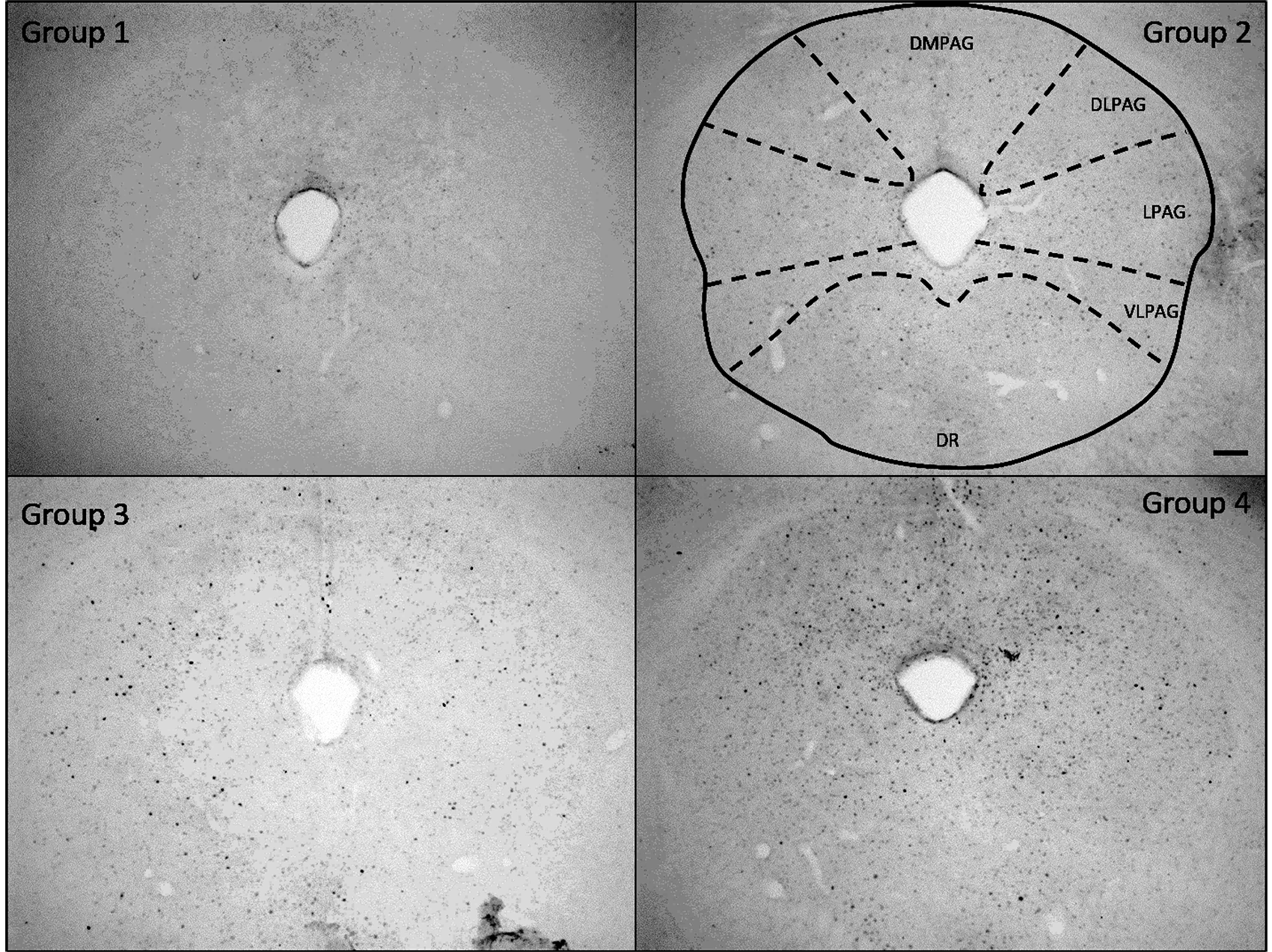

Supplement: S4 Fig — Pictures were taken around -4.36 mm from the Bregma. Scalebar is 100μm. Group1: home cage controls; Group2: no ChR2 controls (transferred for 5 min to the conditioning cage, no ChR2 expression); Group3: no light controls (cage transfer, robust ChR2 expression, not stimulated); Group 4: stimulated by 50Hz theta bursts (cage transfer, robust ChR2 expression, tip of the optical fiber located on the dorso-central part of the MRR (“central”), light administered). DMPAG: dorsomedial part of periaqueductal gray (PAG); DLPAG: dorsolateral PAG; LPAG: lateral PAG; VLPAG: ventrolateral PAG; DR: dorsal raphe nucleus. (TIF) [file pone.0181264.s004.tif]
